# The impact of exposure to cafeteria diet during pregnancy or lactation on offspring growth and adiposity before weaning

**DOI:** 10.1038/s41598-019-50448-x

**Published:** 2019-10-02

**Authors:** Grace George, Sally A. V. Draycott, Ronan Muir, Bethan Clifford, Matthew J. Elmes, Simon C. Langley-Evans

**Affiliations:** School of Biosciences, University of Nottingham, Sutton Bonington Campus, Loughborough, Leicestershire LE12 5RD UK

**Keywords:** Disease model, Obesity

## Abstract

Exposure to maternal obesity during early-life can have adverse consequences for offspring growth and adiposity. We aimed to assess the relative contributions of exposure to maternal obesity, induced by a highly varied cafeteria diet, during pregnancy and lactation on these measures in rat offspring prior to weaning. Female Wistar rats were fed either a control (C) or cafeteria diet (O) for 8 weeks before mating, throughout pregnancy and lactation. Offspring were cross-fostered at birth to a dam on the same (CC,OO) or alternate diet prior to birth (CO,OC). Feeding a cafeteria diet based on 40 different foods, was associated with a sustained period of elevated energy intake before birth and during lactation (up to 1.7-fold), through increased sugar, total fat and saturated fat intake, and lower protein consumption. Cafeteria fed dams sustained greater weight than animals fed a control chow diet and greater perirenal adiposity by the end of lactation. Exposure to obesity during pregnancy was associated with lower offspring birth weight and body weight in early-postnatal life. In contrast, exposure during lactation alone reduced offspring weight but increased adiposity in male CO offspring before weaning. This research highlights that exposure to maternal obesity during lactation alone can programme adiposity in a sex specific manner.

## Introduction

A large body of research suggests that environmental insults during early life, such as maternal obesity, are not only associated with health risks for the mother, but also predispose the developing fetus or infant to risk of non-communicable adult diseases including obesity^1,2^. Adverse health effects for offspring have been identified from studies that have attempted to understand developmental programming mechanisms using small animal models of maternal obesity, through the feeding of high-fat diets from the pre-pregnancy period^3,4^ or at the start of pregnancy, through to the end of lactation^5,6^. Exposure to these diets can lead to remodelling and permanent changes in key offspring organs such as adipose tissue, but these effects are dependent on the composition of the obesogenic diet and the duration and timing of exposure to it, as well as offspring sex and interactions within their postnatal environment^7,8^.

The mechanisms of programming are not fully understood, not least during which early-life windows the developing offspring are more sensitive^9^. The critical developmental window that increases the risk of obesity can be identified through cross-fostering, in which offspring are removed from their birth dam and given to a different dam during suckling. This separates the effects of the prenatal and postnatal environment on offspring health^10^. Cross-fostering studies have already been used to tease apart the adverse metabolic health consequences in offspring exposed to maternal overnutrition before birth^11–13^ and during lactation^11,13–15^. However, the diets used are often high in specific fatty acids and/or sugar, and therefore do not represent the large variation in human overnutrition, or focus specifically on the impact of maternal obesity *per se*. This makes it difficult to identify whether the observed effects are attributed to specific nutrients or maternal obesity. Maternal obesity rather than the diet itself has been demonstrated to be important for programming of offspring adiposity^16^. In addition, exclusively pre-feeding a maternal cafeteria diet to induce maternal obesity in rodent dams before mating, exerted independent effects on offspring growth and metabolic health in adulthood^17^. A cafeteria diet, which ensures a large variety of foods are supplied to induce obesity, gives a better insight into the effects of a maternal obesogenic environment on development^18,19^. The cafeteria diet promotes overfeeding in animal models, because animals have free access to novel, varied and highly palatable and energetically dense human foods, along with standard chow and water^20,21^. This provides an important stimulus for the promotion of hyperphagia in rat dams, leading to obesity during pregnancy and lactation^22^.

Exposure to maternal obesity during early pre and postnatal life via cafeteria feeding has the potential to influence offspring birth weight, growth, body composition, and adiposity in offspring with sex-specific effects^17,23^. One of the main advantages of the cafeteria diet is the variation in foods provided to animals, inducing a sustained overfed state. Previous studies that have used cafeteria diets during pregnancy and lactation have shown limitations in the range of food items provided, varying from six^24^, eight^22^, twelve^17^ and seventeen food items^25^. Therefore, the aim of this study was to establish a more comprehensive and varied cafeteria diet to consider the relative contributions of maternal obesity during pregnancy and lactation on offspring growth and adiposity, using a cross-fostering animal trial design.

## Results

### Maternal weight and food intake

**Figure 1:**
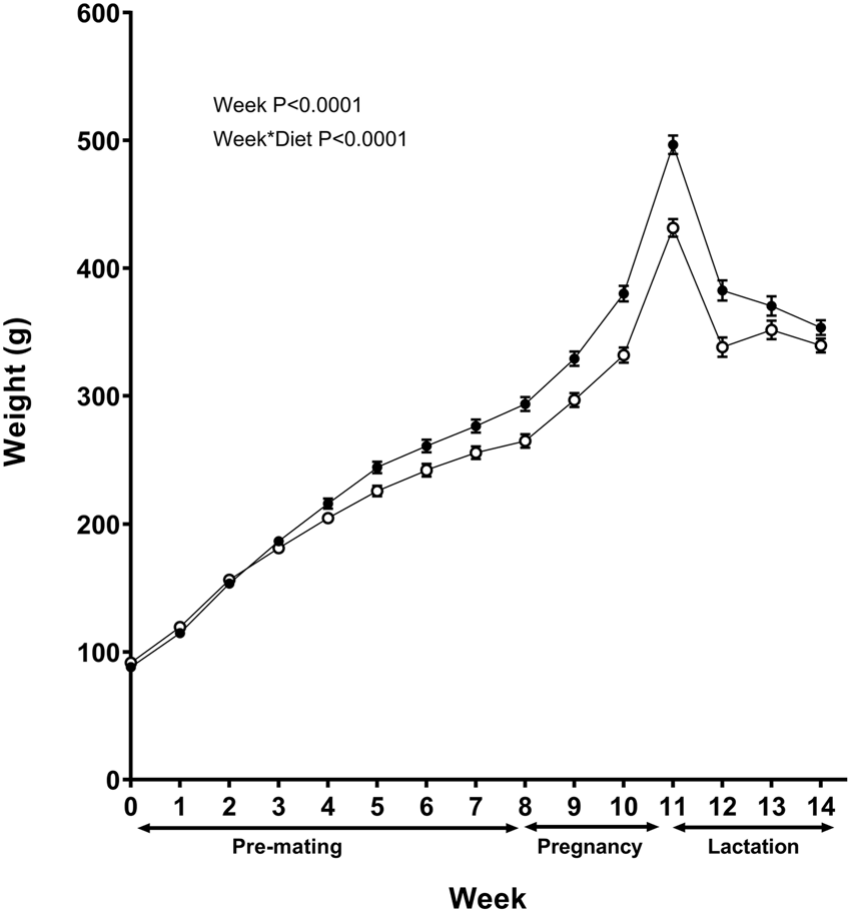
Maternal weight during pre-mating, pregnancy and lactation. Values are for mean and SEM. Animals were fed either a control diet (C, open circles, *n* 13) or a cafeteria diet (O, closed circles, *n* 12). Pre-mating (weeks 0–8), pregnancy (weeks 8–11), lactation (weeks 11–14). Weight gain at week 12 was calculated from the initial lactation weight of female dams after birth. Some animals were excluded from the analysis due to assessment of extreme outliers or data missing. Statistical analysis was by repeated measures ANOVA.

During the first 6 weeks of maternal cafeteria feeding there was no significant variation in weight between rats on their respective diets (Fig. 1). However, from 6–13 weeks on the diet cafeteria fed rats were significantly heavier than control dams. Cafeteria fed rats gained an extra 22% in weight during the pre-pregnancy period compared to controls (O, 205.97 ± 7.57 g versus C, 168.29 ± 4.06 g and significantly greater weight gain was also seen during pregnancy (O, 204.11 ± 3.46 g versus C, 168.28 ± 3.67 g). During weeks 12–14 of lactation the cafeteria fed dams lost weight (−35.01 g ± 6.53 g) compared to weight gain in control dams (31.43 ± 4.43 g). Despite the weight loss, cafeteria fed dams still remained heavier than animals fed a chow diet at mid-lactation (week 13 of the study).

**Figure 2:**
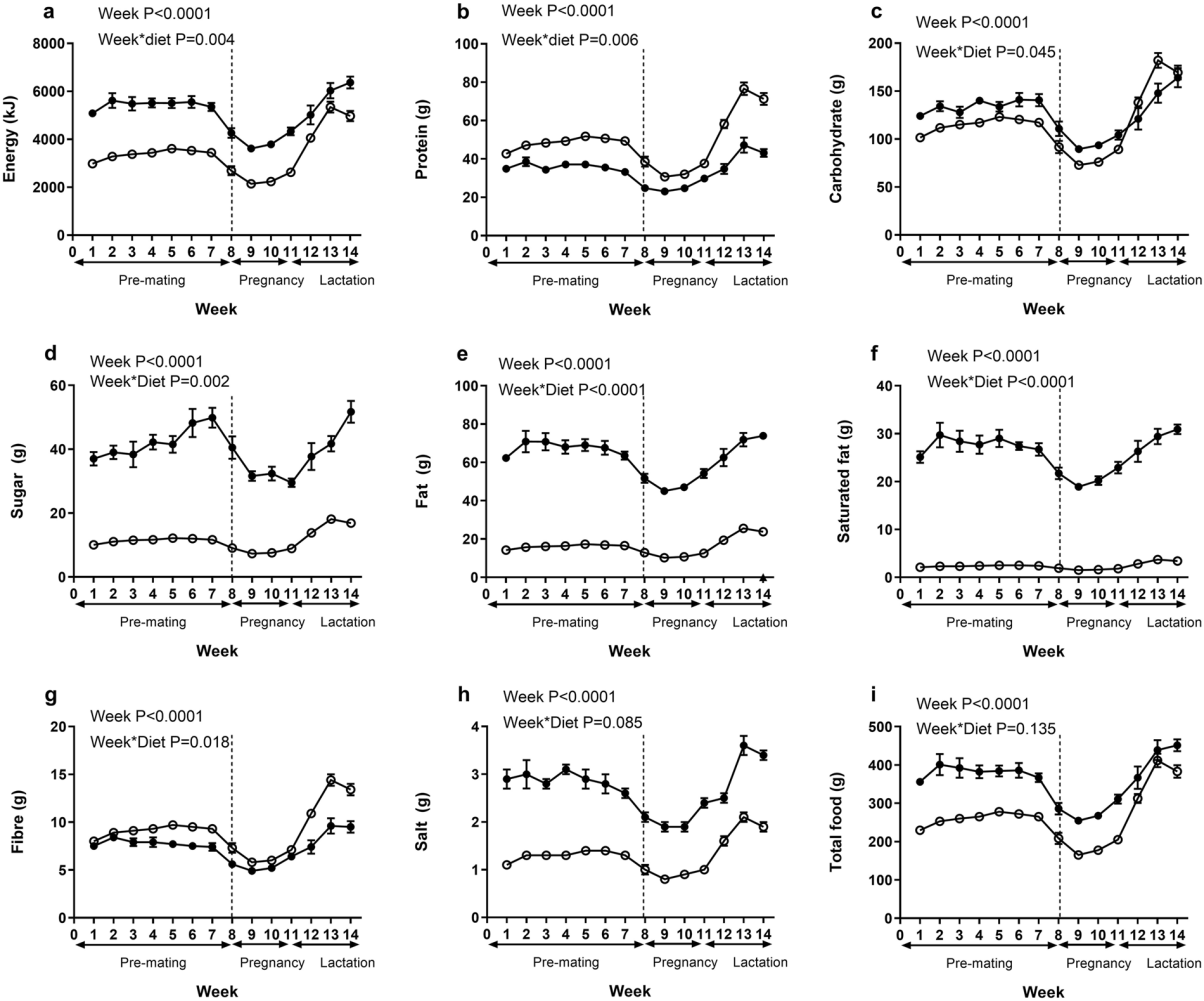
Maternal nutrient intakes. Values are for mean and SEM. Effect of maternal diet on intakes of: (**a**) energy, (**b**) protein, (**c**) carbohydrate, (**d**) sugar, (**e**) fat, (**f**) saturated fat, (**g**) fibre, (**h**) salt, (**i**) total food. Pre-mating (weeks 0–8), pregnancy weeks (8–11), lactation (weeks 11–14). Animals were fed either a control diet (C, open circles *n* 14–16) or a cafeteria diet (O, *closed circles, n* 13–16). Food intakes are shown for total intake of animals pair housed during pre-mating and single-housed during pregnancy and lactation. Pair and single housed data is separated by the dashed line at week 8. Some animals were excluded from the analysis due to assessment of extreme outliers or data missing. Statistical analysis was by repeated measures ANOVA.

Food intake measurements during pre-pregnancy, pregnancy and lactation (Fig. 2) identified that the free choice of foods provided to cafeteria fed dams resulted in them consuming up to 1.6 times greater total food mass and 1.7 times greater energy, over 2 times more salt, 4 times more sugar and fat and 12 times more saturated fat than control dams. Total carbohydrate intake was also significantly higher in cafeteria fed animals, but only up to the end of pregnancy, whereas during lactation intake was higher in chow fed animals. Animals fed the control chow diet demonstrated greater intakes of protein and fibre during the entire phase of feeding. The variation in macronutrient intake reflected the food selections made by the animals, rather than being a factor incorporated into the study design.

**Figure 3:**
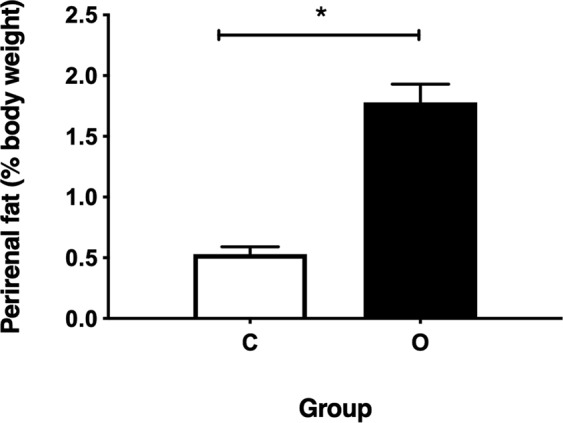
Maternal perirenal adiposity at the end of lactation. Values are for mean and SEM. Animals were fed either a control diet (C, *n* 12) or a cafeteria diet (O, *n* 10). Some animals were excluded from the analysis due to assessment of extreme outliers or data missing. *Effect of maternal diet on maternal perirenal adiposity using an independent samples T-test (*P* < 0.001).

Despite evidence of weight loss in cafeteria fed dams during the lactation period, measurement of perirenal fat mass in the dams at the end of this period demonstrated that they retained 3.4 times greater adiposity, whether expressed as fat per body weight (Fig. 3), or in absolute terms (O 6.43 ± 1.75 g versus C 1.85 ± 0.74 g, *P* < 0.001).

### Offspring birth outcomes, weight and adiposity

**Figure 4:**
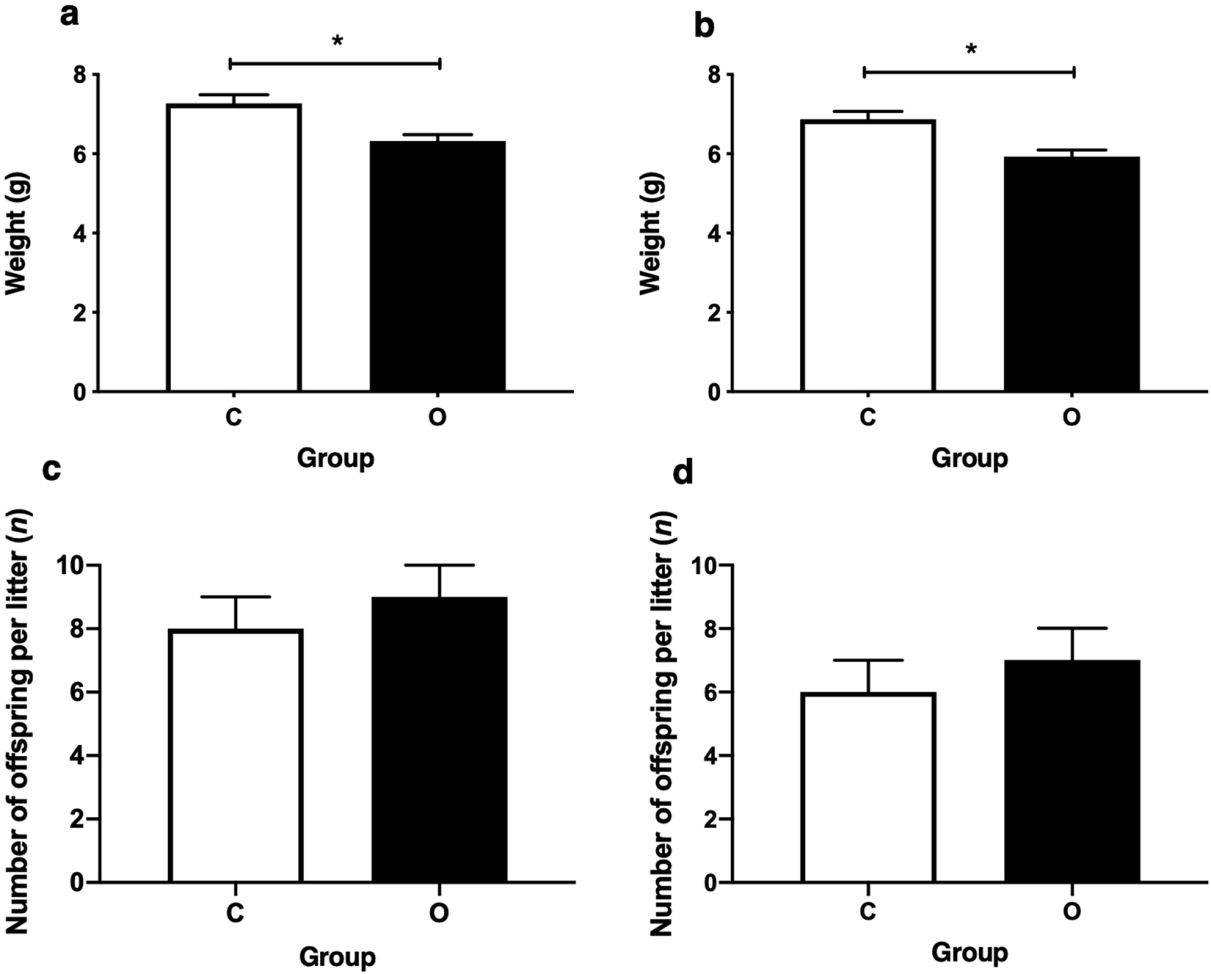
Offspring birth weight and litter size. Values are for mean and SEM. (**a**) Male birth weights, (**b**) female birth weights calculated as the mean per litter, (**c**) male offspring litter size at birth, (**d**) female offspring litter size at birth. Two groups of offspring were studied: offspring exposed to a chow diet during pregnancy (C, *n* 30) or offspring exposed to a cafeteria diet (O, *n* 28). Some animals were excluded from the analysis due to assessment of extreme outliers or data missing. *Effect of maternal pregnancy diet on offspring birth weight using an independent samples T-test for males and females separately (*P* < 0.05). Maternal dietary exposure had no significant effect on the number of offspring per litter for males or females.

**Figure 5:**
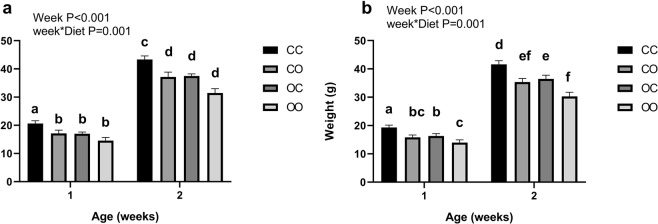
Offspring weekly weights pre-weaning. Values are for are mean and SEM. (**a**) Male offspring, (**b**) Female offspring. Four groups of cross-fostered offspring were studied: offspring exposed to a chow diet during pregnancy cross-fostered to a chow fed dam during lactation (CC, *n* 16) or a cafteria fed dam (CO, *n* 16), offspring exposed to a cafeteria diet during pregnancy cross-fostered to a chow fed dam during lactation (OC, *n* 16) or a cafeteria fed dam (OO, *n* 12). Some animals were excluded from the analysis due to assessment of extreme outliers or data missing. Effect of maternal diet vs time on weekly weight of the offspring was determined through repeated measures ANOVA and Bonferroni post hoc tests. In male offspring, effect of time (*P* < 0.001), Diet*time (*P* = 0.001) and post hoc test revealed CO, OC and OO to be significantly lighter than CC. In female offspring, effect of time (*P* < 0.001), Diet* time (*P* = 0.001), post hoc analysis identified CO, OC and OO to be significantly lighter than CC, and OC to be significantly higher than OO. Different superscript letters signify significant differences at the P < 0.05 level.

There was no significant effect of maternal cafeteria diet on offspring litter size, with the mean number of males (C *n* 8 ± 1, O *n* 9 ± 1) and females (C *n* 6 ± 1, O *n* 7 ± 1) varying little between groups. Exposure to maternal obesity during pregnancy was associated with a significant 13% reduction in offspring birth weights for both males and females, compared to offspring from control fed dams (*P* < 0.05; Fig. 4). In both male and female offspring, exposure to a cafeteria diet during pregnancy and or lactation (CO, OC, and OO) was associated with lower weight than CC at 1 and 2 weeks of age (*P* < 0.05). Interestingly in females only, although there was no difference in birthweight between all cafeteria exposed offspring, OC weighed greater than OO offspring at 1 and 2 weeks of age, suggesting a slower growth rate in OO offspring (Fig. 5).

Analysis of offspring adiposity was initially by 3 way-ANOVA, with pregnancy diet, lactation diet and sex as factors. As sex was identified to have a significant effect Two-way ANOVA was subsequently used for males and females separately as seen in Table 1. This revealed male offspring exposed to maternal obesity during pregnancy (OO, OC) had less perirenal adipose tissue at 2 weeks of age (P = 0.005), whereas male CO offspring had 40% greater perirenal fat mass than CC offspring (P = 0.043). In contrast, the level of gonadal fat was unaffected by dietary treatment in male offspring. Female offspring had less fat at both sites than males (P < 0.05), and neither depot showed any response to maternal obesity or diet.

**Table 1:** Offspring adipose tissue mass at 2 weeks of age.

Gender	Group	Perirenal fat mass (% body weight)		Gonadal fat mass (% body weight)	
		Mean	SEM	Mean	SEM
Male	CC	0.30	0.02	0.15	0.01
	CO	^b^0.42	0.04	0.14	0.01
	OC	^a^0.28	0.02	0.13	0.01
	OO	^a^0.29	0.02	0.15	0.02
Female	CC	0.21	0.03	0.15	0.02
	CO	0.26	0.02	0.23	0.03
	OC	0.20	0.02	0.14	0.02
	OO	0.18	0.05	0.16	0.01

Values are for mean and SEM. Four groups of cross-fostered offspring were studied: offspring exposed to a chow diet during pregnancy cross-fostered to a chow fed dam during lactation (CC, *n* 15–16) or a cafteria fed dam (CO, *n* 12–16), offspring exposed to a cafeteria diet during pregnancy cross-fostered to a chow fed dam during lactation (OC, *n* 16) or a cafeteria fed dam (OO, *n* 9–12). Some animals were excluded from the analysis due to assessment of extreme outliers or data missing.

^a^Effect of maternal pregnancy diet on offspring perirenal adiposity using two-way ANOVA (males only) (*P* = 0.005).

^b^Interaction effect of maternal pregnancy and maternal lactation diet on offspring perirenal adiposity using two-way ANOVA (males only) (*P* = 0.043).

## Discussion

The current study was successful in identifying the relative contributions of maternal obesity during pregnancy and lactation on offspring growth and adiposity, by utilising a highly varied and palatable cafeteria diet to induce maternal obesity, rather than feeding diets high in specific fatty acids or sugars^11–15^ during critical periods of development. The greater range of novel foods used in this study produced a more prolonged impact on food intake and adiposity than previous cafeteria diet protocols^17,18^.The data suggests that exposure to maternal obesity during the suckling period had a greater influence on male offspring adiposity pre-weaning (CO versus CC).

Maternal nutritional intakes of the cafeteria diet were relatively comparable to previous studies that also selected a cafeteria diet to induce maternal obesity, including higher intakes of energy, fat, and lower intakes of protein^17,22,25^. However, the present study used a cafeteria diet that comprised of 40 highly palatable energy-dense human food items providing one of the most varied diets ever reported for a rodent study. Previous studies that have used fewer food items have reported less maternal weight gain and sustained nutrient intakes in cafeteria fed dams^17,18^, or have reported no significant differences in body weights of control or cafeteria fed dams^26^. The cafeteria diet in this study was successful at inducing maternal obesity, evidenced by maintained interest through consistently greater intakes of high energy food, leading to sustained hyperphagia and greater weight gain, and over 3 times greater perirenal adiposity at the end of lactation compared to control fed dams. This was an important observation as it has been demonstrated that maternal obesity, rather than the maternal diet, is necessary for programming of offspring obesity and metabolism^16,17^. Although maternal weight and maternal perirenal adiposity at the end of lactation were the only measures of maternal obesity, female dams fed a cafeteria diet have been shown to have greater total body adiposity during both early and late pregnancy^18,25^. Animals remained obese up to the end of lactation, despite weight loss during this time. Weight loss during lactation could be due to increased fat deposition during pregnancy to provide greater energy reserves for maintaining metabolic demand during lactation^27^, or it could be that weight loss was due to mobilisation of lean mass^25^, which would require future investigation.

Offspring born to cafeteria fed dams exhibited significantly lower birth weights when compared to offspring of control dams. Exposure to a maternal cafeteria diet has been shown to reduce fetal weight of offspring at day 20 of pregnancy, which was dependent on the pre-pregnancy maternal cafeteria diet and therefore maternal obesity rather than the cafeteria diet itself^18^. Reduced fetal and placental weight^25^ and birth weight^22,26,28^ have been demonstrated in other studies that used a cafeteria diet as a model of maternal obesity. Together, these data suggest that maternal obesity in rodents leads to intrauterine growth restriction and reduced birth weight in offspring, although some studies have indicated no differences between birth weight of offspring^29^ or increased birth weight^17,30^. In humans, maternal obesity is mainly associated with infants that are large-for-gestational age^31,32^. Therefore, it appears that variations exist between species, and is dependent on the timing and type of exposure to maternal overnutrition.

Exposure to maternal obesity was associated with lower offspring weights during early postnatal life, possibly due to a failure to catch up from lower birthweight. However exposure during suckling appeared to have an additive effect as OO offspring were lightest, consistent with findings by Vithayathil *et al*.^28^, who used a similar trial protocol. This is counter-intuitive as the excess energy intake of the dams may be expected to result in enriched milk (energy from fat), stimulating greater growth. Reduced weight at birth up to 2 weeks of age could be associated with the low protein composition of the cafeteria diet, or a reduction in micronutrient intakes. Exposure to maternal low protein diets during early life is associated with programming of offspring growth and adiposity^33^. However, it has been shown that the milk protein content in stomachs of offspring at birth did not differ between those exposed to a maternal cafeteria diet or control chow diet during pregnancy, nor did the protein in the milk of lactating cafeteria fed dams differ from control fed dams^34^. Instead, altered concentrations of fatty acids that can influence development were found in offspring exposed to a cafeteria diet, including higher levels of monounsaturated and trans fatty acids, as well as lower n-3 long chain polyunsaturated fatty acids^34^. In addition, maternal obesity, rather than the cafeteria diet itself, has been associated with lower offspring weights throughout life^17^. The suggestion that the effect of diet on health does not arise from a single nutrient, but a combination of nutrients working in synergy is why the focus on dietary patterns is more advantageous when looking at diet disease relationships^35^. A varied cafeteria diet high in saturated fats, sugar, and salt, may have a combined effect on offspring health, given that feeding dams these individual nutrients during pregnancy and lactation is associated with altered offspring physiology and metabolism^36–38^.

Male offspring exposed to maternal obesity during lactation alone demonstrated elevated perirenal adiposity at 2 weeks of age, despite lighter body weights. Postnatal exposure to maternal high-fat feeding has previously been reported to influence male and female offspring visceral adiposity and growth during the early stages of life, before weaning, but this effect was lost post-weaning^14,28^. The opposite effect was seen in male offspring exposed to maternal obesity before birth as well (OO), suggesting that they could be biologically prepared for an adverse maternal environment that may give them an adaptive advantage^39^. Interestingly, these effects were not seen in female offspring or in the gonadal adipose depots. It is known that exposure to maternal obesity during lactation can programme for depot and sex-specific effects on adipose tissue development^40^. Future research would be required to understand the mechanisms behind this, such as interaction with nutritional signals and sex-hormones^41^, or epigenetic modifications^42^.

There are strong associations between reduced weight at birth and during early-life and rapid catch up growth and disease in adult life, including obesity and insulin resistance, in humans^43,44^ and rats^45^. It is becoming increasingly clear that such patterns of growth are as strongly associated with maternal overnutrition as with undernutrition. The present study highlights that exposure to maternal obesity impairs fetal growth in the rat, and that obesity and a cafeteria diet during suckling increases pre-weaning adiposity in male, but not female offspring. This study has demonstrated a robust and novel protocol for inducing prolonged overfeeding in rats which can be used to examine the separate influences of obesity in pregnancy and lactation. The presence of sex-specific effects is intriguing as they precede pubertal changes in body composition and suggest that the perinatal period is a time when preadipocyte development is under the influence of sex-related factors and maternal diet, adiposity and possibly endocrine signalling. There are some unanswered questions that would warrant future investigation, including analysis of the milk composition of lactating cafeteria dams. This study has provided a strong demonstration that maternal obesity drives differential effects in pregnancy and lactation, which are likely to have long-term impacts on offspring health and wellbeing.

## Methods

### Animal procedures

All animal procedures were performed in accordance with the Animals (Scientific Procedures) Act 1986 under Home Office licence and were approved by the Animal Ethics Committee of the University of Nottingham, UK. Animals were subjected to a controlled 12-hour light, 12-hour dark cycle, in conditions at 20–22 °C and 45% humidity. They were housed in plastic cages with environmental enrichment and had *ad libitum* access to food and water. Virgin female Wistar rats (Charles River, UK), four weeks old (approximately 95 g; *n* 32) were randomly allocated to be fed either a control chow diet (C; *n* 16) (Teklad Global 18% Rodent Diet, Harlan, Belton, now Envigo) or a cafeteria diet (O; *n* 16). The cafeteria diet consisted of 40 different highly palatable, energy-rich human foods, accompanied by laboratory chow. Four foods in excess quantities ( > 10 g) were randomly provided daily to each animal in a bowl on the cage floor. To maintain variety, two food items were replaced daily, so animals did not receive the same items for more than two consecutive days. The list of cafeteria diet food items with nutritional information is listed in Supplementary Table S1. Animal weights and food intake were measured daily and nutritional intakes (energy, fat, saturated fat, total carbohydrate, sugar, fibre, salt and protein) were calculated as weekly intakes, based on manufacturer’s data. Animals were fed their respective diets for 8 weeks prior to mating through pregnancy and during lactation. In the pre-mating period, animals were pair housed. At mating, all rats were housed with Wistar stud males and mating confirmed by the appearance of a semen plug. Animals were then singly housed and remained on their respective diets throughout pregnancy and lactation. Dams were weighed after birth for calculation of weight gain/loss the following week.

### Cross-fostering and offspring weights

All offspring were cross-fostered within 96 hours of birth. Four groups of cross-fostered offspring were generated: offspring exposed to a chow diet during pregnancy cross-fostered to a chow-fed dam during lactation (CC; *n* 64) or a cafeteria-fed dam (CO; *n* 64), offspring exposed to a cafeteria diet during pregnancy cross-fostered to a chow-fed dam during lactation (OC; *n* 64) or a cafeteria-fed dam (OO; *n* 48). Cross-fostering was achieved by removing the dam from their offspring and placing them in a separate cage out of view of the procedure. Using clean gloves to avoid smell of the birth dam, offspring were then removed from the cage. Offspring were weighed, sexed and randomly allocated to be used or culled so that litters consisted of a maximum of 8 pups (4 females and 4 males where possible). Without disturbing the nest, fostered offspring were then positioned in the cage of the foster mother in the same location as offspring removed. Up to 4 litters were cross-fostered at a time because the experiment was staggered so that only 4 female rats were introduced to the protocol per week, this led to only 4 dams giving birth per week. Offspring were observed regularly to ensure acceptance of offspring through the presence of suckling. Offspring were weighed weekly from cross fostering.

### Tissue collection

Neonates were culled using cervical dislocation and cessation of the circulation. Dams and older offspring were culled using CO_2_ asphyxia and cervical dislocation. All tissue collected throughout the study was stored in 1.5 mL tubes that were snap frozen in liquid nitrogen before being stored at −80 °C. Perirenal and gonadal adipose tissue was weighed and collected at 2 weeks of age from one randomly selected male and one randomly selected female pup. Perirenal adipose tissue was collected from culled female dams at the end of lactation. Tissue weights were calculated as a percentage of animal body weight.

### Statistical analysis

All data was analysed using Statistical Package for Social Sciences (version 22; SPSS, Inc., Chicago, IL, USA). All data was checked for normal distribution using the Shapiro-Wilk test of normality and normality plots. If data was not normally distributed, data was transformed using the appropriate method based on the skewness of the data. For the effect of maternal diet on maternal food intake, maternal weight and offspring weight at 1 and 2 weeks of postnatal age, a repeated measures ANOVA was used. An independent samples T-test was also used to investigate the effect of maternal diet on maternal adiposity and total weight gain during the individual pre-pregnancy, pregnancy, and lactation periods. An independent samples T-test was also performed separately for males and females, to investigate the effect of maternal diet on offspring litter size and offspring birth weight due to sex differences in these outcomes. Due to sex differences between male and female offspring weights at week 1 and 2 and offspring adiposity, as determined by the three-way ANOVA, differences between groups were assessed separately in male and female offspring. A general linear model two-way ANOVA was used to measure the effect of maternal obesity on offspring adiposity (fixed factors were maternal pregnancy diet and maternal lactation diet). To investigate the effect of maternal diet on offspring weight at week 1 and 2, a repeated measures ANOVA was used and a Bonferroni post hoc test used to identify significance between groups. For offspring weights, where there was more than one offspring per litter, means per litter were identified and then statistical analysis was performed with this data. This was calculated from all offspring at birth, and from a litter size of 8 at weeks 1 and 2. *P* < 0.05 was considered statistically significant. Variable *n* numbers presented in the results, mean that some animals were excluded from some analyses due to extreme outliers or data missing. Two cafeteria fed dams and their litters were excluded from the trial after pregnancy, one due a pregnancy complication and one due to cannibalism.

### Ethical approval and informed consent

This study was approved by the Animal Ethics Committee of the University of Nottingham, UK. All animal procedures were performed in accordance with the Animals (Scientific Procedures) Act 1986 under Home Office licence.

## Supplementary information

Supplementary Table S1. List of cafeteria diet food items and nutritional information.

Supplementary Table 2. Example of a cafeteria diet feeding regime over a 14 week period.

## Data Availability

The datasets generated during and/or analysed during the current study are available from the corresponding author on reasonable request.
