# Supplementary material for: The impact of exposure to cafeteria diet during pregnancy or lactation on offspring growth and adiposity before weaning
Source: Sci Rep. 2019 Oct 2;9:14173. doi: 10.1038/s41598-019-50448-x (PMC6775089; doi:10.1038/s41598-019-50448-x)
Supplement: Supplementary file 1 — Supplementary Table S1. List of cafeteria diet food items and nutritional information. [file 41598_2019_50448_MOESM1_ESM.docx]

**The impact of exposure to cafeteria diet during pregnancy or lactation on offspring growth and adiposity before weaning.**

Grace George^1^, Sally A.V. Draycott^1^, Ronan Muir^1^, Bethan Clifford^1^, Matthew J. Elmes^1^, Simon C. Langley-Evans^1^. ^1^School of Biosciences, University of Nottingham, Sutton Bonington Campus, Loughborough, Leicestershire, LE12 5RD, UK.

Corresponding Author. Dr Matthew Elmes,

Email: matthew.elmes@nottingham.ac.uk

| **Food item** | **Energy (kcal)** | **Protein (g)** | **Carbohydrate (g)** | **Sugars (g)** | **Fat (g)** | **Saturated fat (g)** | **Fibre (g)** | **Salt (g)** |
| --- | --- | --- | --- | --- | --- | --- | --- | --- |
| Chow diet | 310 | 18.6 | 44.2 | 4.4 | 6.2 | 0.9 | 3.5 | 0.5 |
| **Crisps and snacks** |  |  |  |  |  |  |  |  |
| Doritos BBQ tortillas (200g) | 493 | 6.2 | 56 | 3.4 | 25.6 | 2.1 | 6.9 | 1.78 |
| Doritos lime tortillas (200g) | 498 | 6.2 | 56.5 | 1.8 | 25.9 | 2.2 | 7 | 1.25 |
| Jacob's crispy baked cheddars crispy bacon (150g) | 531 | 10.8 | 48.5 | 5.4 | 31.8 | 15.7 | 3 | 1.6 |
| Tesco everyday value ready salted crisps (12 pack) | 553 | 5 | 52.8 | 0.7 | 32.7 | 3.3 | 3.8 | 1 |
| Tesco prawn crackers (40g) | 550 | 2.2 | 60.4 | 6.8 | 33.2 | 3.4 | 0.6 | 2.2 |
| **Cakes and confectionery** |  |  |  |  |  |  |  |  |
| Mr Kipling trifle Bakewells (6 Pack) | 422 | 3.7 | 62.5 | 36.9 | 17.3 | 6.9 | 0.9 | 0.31 |
| Pink Panther Wafers (200g) | 550 | 5.4 | 59.4 | 26.3 | 32 | 20 | 1.4 | 0.2 |
| Lees 6 chocolate teacakes (110g) | 449 | 3.2 | 72.3 | 49 | 15.8 | 12.4 | 2.7 | 0.2 |
| Tesco double chocolate Swiss roll (192g) | 396 | 5.1 | 63.2 | 32.4 | 13.3 | 6.4 | 1.5 | 0.6 |
| Tesco everyday value raspberry flavoured sponge cake (228g) | 353 | 4.1 | 59.8 | 36.9 | 10.4 | 3 | 2.1 | 0.5 |
| Tesco strawberry swirl cheesecake (375g) | 320 | 4.9 | 41.9 | 23.9 | 14.6 | 4.7 | 0.9 | 0.3 |
| Tesco assorted jam tarts (6 pack) | 408 | 3.4 | 67.3 | 37.2 | 13.6 | 6.4 | 1.5 | 0.2 |
| Tesco everyday value cherry Bakewells (6 pack) | 411 | 3.9 | 65.2 | 40.3 | 14.7 | 6.4 | 1.2 | 0.2 |
| Tesco chocolate malted milk biscuits (250g) | 506 | 6.4 | 64.5 | 29.6 | 24.2 | 12.4 | 2.1 | 0.7 |
| Tesco everyday value milk chocolate digestives (300g) | 494 | 7.2 | 65.4 | 39.2 | 22.3 | 11.1 | 1.7 | 1 |
| Tesco everyday value mince pies (6 pack) | 411 | 4 | 60.3 | 23.2 | 16.4 | 7.4 | 2.9 | 0.1 |
| Tesco everyday value chocolate and vanilla Swiss Roll (104g) | 383 | 5.5 | 59.3 | 31.4 | 13.4 | 6.4 | 1.7 | 0.6 |
| Tesco marshmallows (200g) | 332 | 4.1 | 77.7 | 64.9 | 0.4 | 0.2 | 0.5 | 0.1 |
| Tesco mini Strawberry doughnuts (18 pack) | 401 | 5.3 | 50.8 | 27.4 | 19.2 | 10.6 | 2.2 | 0.4 |
| Tesco peanut butter milk chocolate (150g) | 553 | 8 | 53.1 | 50.5 | 33.8 | 18.5 | 2.2 | 0.4 |
| Mcvitie's golden syrup cake (223g) | 355 | 3.6 | 60.5 | 37.8 | 10.8 | 3.3 | 1.6 | 1 |
| Mcvitie's Jaffa cakes | 380 | 4.9 | 70.8 | 52.5 | 8 | 4.1 | 2.2 | 0.24 |
| Mcvitie's Jamaica ginger cake (223g) | 364 | 3.7 | 63.4 | 37.8 | 10.5 | 3.2 | 1.6 | 0.5 |
| Tesco mini chocolate doughnuts (18 pack) | 430 | 4.8 | 48 | 22.9 | 23.9 | 12.3 | 2.1 | 0.4 |
| Tesco brioche chocolate chip rolls (8 pack) | 385 | 7.9 | 52.1 | 18.5 | 15.6 | 8.2 | 2.5 | 0.8 |
| Tesco custard cream biscuits (400g) | 493 | 5.6 | 69.7 | 28.5 | 20.9 | 10.7 | 1.7 | 0.5 |
| **Savoury items** |  |  |  |  |  |  |  |  |
| Tesco 2 pepperoni pizza slices (300g) | 292 | 7.3 | 20.3 | 2.1 | 19.6 | 9.2 | 2.7 | 0.7 |
| Tesco 20 processed cheese slices (400g) | 248 | 12 | 12.9 | 8.9 | 16.5 | 9.8 | 0 | 2 |
| Tesco 4 cheese and onion pasties (520g) | 269 | 7.3 | 21.9 | 2.1 | 16.4 | 8.4 | 2.3 | 0 |
| Tesco cheese and onion quiche (400g) | 279 | 9 | 19.5 | 2 | 17.9 | 7.8 | 1.7 | 0.4 |
| Tesco mini savoury eggs (216g) | 293 | 9.5 | 19.2 | 0.6 | 19.4 | 5.5 | 1.7 | 0.8 |
| Tesco everyday value mature cheddar cheese (1kg) | 416 | 25.4 | 0.1 | 0.1 | 34.9 | 21.7 | 0 | 1.8 |
| **Meat** |  |  |  |  |  |  |  |  |
| Tesco smoky bacon cocktail bitesize sausages (255g) | 277 | 13.8 | 12.1 | 1.8 | 18.9 | 7 | 1.7 | 1.7 |
| Mattesson’s smoked pork sausage original (200g) | 310 | 15 | 0 | 0 | 29 | 13 | 0 | 2.2 |
| Tesco 6 mini Melton Mowbray pork pies (300g) | 383 | 10.6 | 26.9 | 1.9 | 25.6 | 9.9 | 1.5 | 1.1 |
| Tesco everyday value 8 sausage rolls (480g) | 316 | 7.4 | 31.7 | 1.3 | 17.2 | 7.5 | 2.5 | 1 |
| Tesco bitesize Lincolnshire cocktail sausages (255g) | 284 | 13.1 | 10.8 | 1 | 20.6 | 7.3 | 1.6 | 1.5 |
| Tesco Brussels pate (200g) | 352 | 14 | 3.3 | 2.8 | 31.2 | 12.7 | 0.9 | 2 |
| Tesco everyday value pork pies (440g) | 372 | 9 | 25.7 | 3.1 | 25.3 | 10.7 | 2.7 | 1.5 |
| Tesco 70 cocktail sausages (616g) | 257 | 12.9 | 12.8 | 1.5 | 16.7 | 6.1 | 2 | 1.5 |
|  |  |  |  |  |  |  |  |  |
| **Supplementary Table S1. List of cafeteria diet food items and nutritional information.** | | | | | | | | |
| Food items fed to cafeteria dams throughout pre-mating, pregnancy and lactation. Nutritional values are from manufacturer’s data. | | | | | | | |  |
