# Supplementary material for: The impact of exposure to cafeteria diet during pregnancy or lactation on offspring growth and adiposity before weaning
Source: Sci Rep. 2019 Oct 2;9:14173. doi: 10.1038/s41598-019-50448-x (PMC6775089; doi:10.1038/s41598-019-50448-x)
Supplement: Supplementary file 2 — Supplementary Table 2. Example of a cafeteria diet feeding regime over a 14 week period. [file 41598_2019_50448_MOESM2_ESM.docx]

**The impact of exposure to cafeteria diet during pregnancy or lactation on offspring growth and adiposity before weaning.**

Grace George^1^, Sally A.V. Draycott^1^, Ronan Muir^1^, Bethan Clifford^1^, Matthew J. Elmes^1^, Simon C. Langley-Evans^1^. ^1^School of Biosciences, University of Nottingham, Sutton Bonington Campus, Loughborough, Leicestershire, LE12 5RD, UK.

Corresponding Author. Dr Matthew Elmes,

Email: [matthew.elmes@nottingham.ac.uk](mailto:matthew.elmes@nottingham.ac.uk)

| **Week** | **Cafeteria diet food combinations** | | | |
| --- | --- | --- | --- | --- |
|  | **Food item 1** | **Food item 2** | **Food item 3** | **Food item 4** |
| 1 | Chocolate swiss roll | Pepperoni pizza slices | Jaffa cakes | Crispy bacon baked cheddars |
|  | Chocolate swiss roll | Pepperoni pizza slices | Processed cheese slices | Strawberry cheesecake |
|  | Brussels pate | Golden syrup cake | Processed cheese slices | Strawberry cheesecake |
|  | Jam tarts | Mini Strawberry doughnuts | Chocolate Teacakes | Pork pie |
|  | Cheese & onion quiche | Mini savoury eggs | Smoked pork sausage | Processed cheese slices |
| 2 | Cheese & onion quiche | Mini savoury eggs | Smoked pork sausage | Processed cheese slices |
|  | Jaffa cakes | Prawn crackers | Custard Creams | Tortillas |
|  | Jaffa cakes | Prawn crackers | Mature Cheddar | Smokey bacon cocktail sausages |
|  | Peanut butter chocolate | Trifle Bakewell | Mature Cheddar | Smokey bacon cocktail sausages |
|  | Pepperoni pizza slices | Golden syrup cake | Mini chocolate doughnuts | Melton Mowbray Pork Pies |
| 3 | Chocolate swiss roll | Processed cheese slices | Chocolate chip brioche | Ready salted crisps |
|  | Chocolate swiss roll | Processed cheese slices | Sausage rolls | Pink wafers |
|  | Jam tarts | Brussels pate | Sausage rolls | Pink wafers |
|  | Jam tarts | Brussels pate | Pepperoni pizza slices | Marshmallows |
|  | Trifle Bakewell | Cheese & onion quiche | Peanut butter chocolate | Chocolate malted milk |
| 4 | Mature Cheddar | Prawn crackers | Chocolate Teacakes | Raspberry sponge cake |
|  | Cheese & onion pasties | Lincolnshire cocktail sausages | Chocolate Teacakes | Raspberry sponge cake |
|  | Cheese & onion pasties | Lincolnshire cocktail sausages | Marshmallows | Chocolate digestives |
|  | Mini savoury eggs | Melton Mowbray Pork Pies | Marshmallows | Chocolate digestives |
|  | Pink wafers | Sausage rolls | Processed cheese slices | Mini Strawberry doughnuts |
| 5 | Mini savoury eggs | Jam tarts | Melton Mowbray Pork Pies | Chocolate swiss roll |
|  | Strawberry cheesecake | Brussels pate | Melton Mowbray Pork Pies | Chocolate swiss roll |
|  | Strawberry cheesecake | Brussels pate | Pepperoni pizza slices | Trifle Bakewell |
|  | Marshmallows | Ready salted crisps | Pepperoni pizza slices | Trifle Bakewell |
|  | Cheese & onion quiche | Mini savoury eggs | Jamaican ginger cake | Raspberry sponge cake |
| 6 | Mature Cheddar | Trifle Bakewell | Prawn crackers | Jam tarts |
|  | Cocktail sausages | Chocolate Teacakes | Prawn crackers | Jam tarts |
|  | Cocktail sausages | Chocolate Teacakes | Cheese & onion pasties | Mini Strawberry doughnuts |
|  | Mince pies | Processed cheese slices | Cheese & onion pasties | Mini Strawberry doughnuts |
|  | Smoked pork sausage | Pepperoni pizza slices | Marshmallows | Custard Creams |
| 7 | Chocolate swiss roll | Ready salted crisps | Melton Mowbray Pork Pies | Pink wafers |
|  | Chocolate swiss roll | Ready salted crisps | Brussels pate | Mince pies |
|  | Raspberry sponge cake | Prawn crackers | Brussels pate | Mince pies |
|  | Raspberry sponge cake | Prawn crackers | Processed cheese slices | Chocolate Teacakes |
|  | Cheese & onion pasties | Cocktail sausages | Mini chocolate doughnuts | Chocolate chip brioche |
| 8 | Trifle Bakewell | Processed cheese slices | Smoked pork sausage | Golden syrup cake |
|  | Trifle Bakewell | Processed cheese slices | Strawberry cheesecake | Sausage rolls |
|  | Brussels pate | Marshmallows | Strawberry cheesecake | Sausage rolls |
|  | Brussels pate | Marshmallows | Prawn crackers | Pink wafers |
|  | Raspberry sponge cake | Mini savoury eggs | Custard Creams | Pork pie |
| 9 | **Includes Mating Period** | | | |
|  | Cheese & onion pasties | Chocolate swiss roll | Cheese & onion quiche | Mini Strawberry doughnuts |
|  | Cheese & onion quiche | Mini Strawberry doughnuts | Processed cheese slices | Custard Creams |
|  | Mature Cheddar | Sausage rolls | Mince pies | Chocolate Teacakes |
| 10 | Marshmallows | Mini savoury eggs | Pepperoni pizza slices | Chocolate chip brioche |
|  | Marshmallows | Mini savoury eggs | Mini chocolate doughnuts | Pork pie |
|  | Trifle Bakewell | Ready salted crisps | Mini chocolate doughnuts | Pork pie |
|  | Trifle Bakewell | Ready salted crisps | Brussels pate | Pink wafers |
|  | Cheese & onion quiche | Strawberry cheesecake | Mature Cheddar | Jamaican ginger cake |
| 11 | Cocktail sausages | Cheese & onion pasties | Chocolate chip brioche | Jam tarts |
|  | Cocktail sausages | Cheese & onion pasties | Mince pies | Chocolate and vanilla swiss roll |
|  | Mini savoury eggs | Sausage rolls | Mince pies | Chocolate and vanilla swiss roll |
|  | Mini savoury eggs | Sausage rolls | Cherry Bakewell | Pink wafers |
|  | Mini Strawberry doughnuts | Processed cheese slices | Pork pie | Chocolate chip brioche |
| 12 | Raspberry sponge cake | Smoked pork sausage | Ready salted crisps | Marshmallows |
|  | Raspberry sponge cake | Smoked pork sausage | Pepperoni pizza slices | Chocolate Teacakes |
|  | Custard Creams | Cocktail sausages | Pepperoni pizza slices | Chocolate Teacakes |
|  | Custard Creams | Cocktail sausages | Brussels pate | Jam tarts |
|  | Cherry Bakewell | Chocolate chip brioche | Mini savoury eggs | Cheese & onion pasties |
| 13 | Processed cheese slices | Pork pie | Mini Strawberry doughnuts | Golden syrup cake |
|  | Processed cheese slices | Pork pie | Chocolate and vanilla swiss roll | Mince pies |
|  | Smoked pork sausage | Ready salted crisps | Chocolate and vanilla swiss roll | Mince pies |
|  | Smoked pork sausage | Ready salted crisps | Marshmallows | Chocolate Teacakes |
|  | Strawberry cheesecake | Cheese & onion quiche | Cocktail sausages | Custard Creams |
| 14 | Raspberry sponge cake | Mini savoury eggs | Mature Cheddar | Chocolate chip brioche |
|  | Raspberry sponge cake | Mini savoury eggs | Sausage rolls | Chocolate Teacakes |
|  | Golden syrup cake | Processed cheese slices | Sausage rolls | Chocolate Teacakes |
|  | Golden syrup cake | Processed cheese slices | Jam tarts | Ready salted crisps |
|  | Brussels pate | Cherry Bakewell | Mince pies | Smoked pork sausage |
| **Supplementary Table 2. Example of a cafeteria diet feeding regime over a 14 week period.** At least two food items were replaced daily where possible to maintain variety, except for at weekends where animals received foods for consecutive days. Animals were fed a chow diet in combination with a cafeteria diet. During mating, animals were only fed a chow diet. | | | | |
|  |  |  |  |  |
|  |  |  |  |  |
|  |  |  |  |  |
